# Supplementary material for: Identification and RNAi Profile of a Novel Iflavirus Infecting Senegalese Aedes vexans arabiensis Mosquitoes
Source: Viruses. 2020 Apr 14;12(4):440. doi: 10.3390/v12040440 (PMC7232509; doi:10.3390/v12040440)

Figure S1: **Assembly strategy of Aedes vexans iflavirus (AvIFV) genome** A) Genome schematic of AvIFV B) AvIFV contigs generated using SPAdes [1] under different assembler flags C) AvIFV genome contigs assembled using Trinity v2.1.1 [2] under default conditions and D) AvIFV genome contigs assembled using CLC Genomics workbench (QIAGEN Aarhus A/S). Overlapping regions are shown in **bold**, and corresponding gRNA location are indicated.

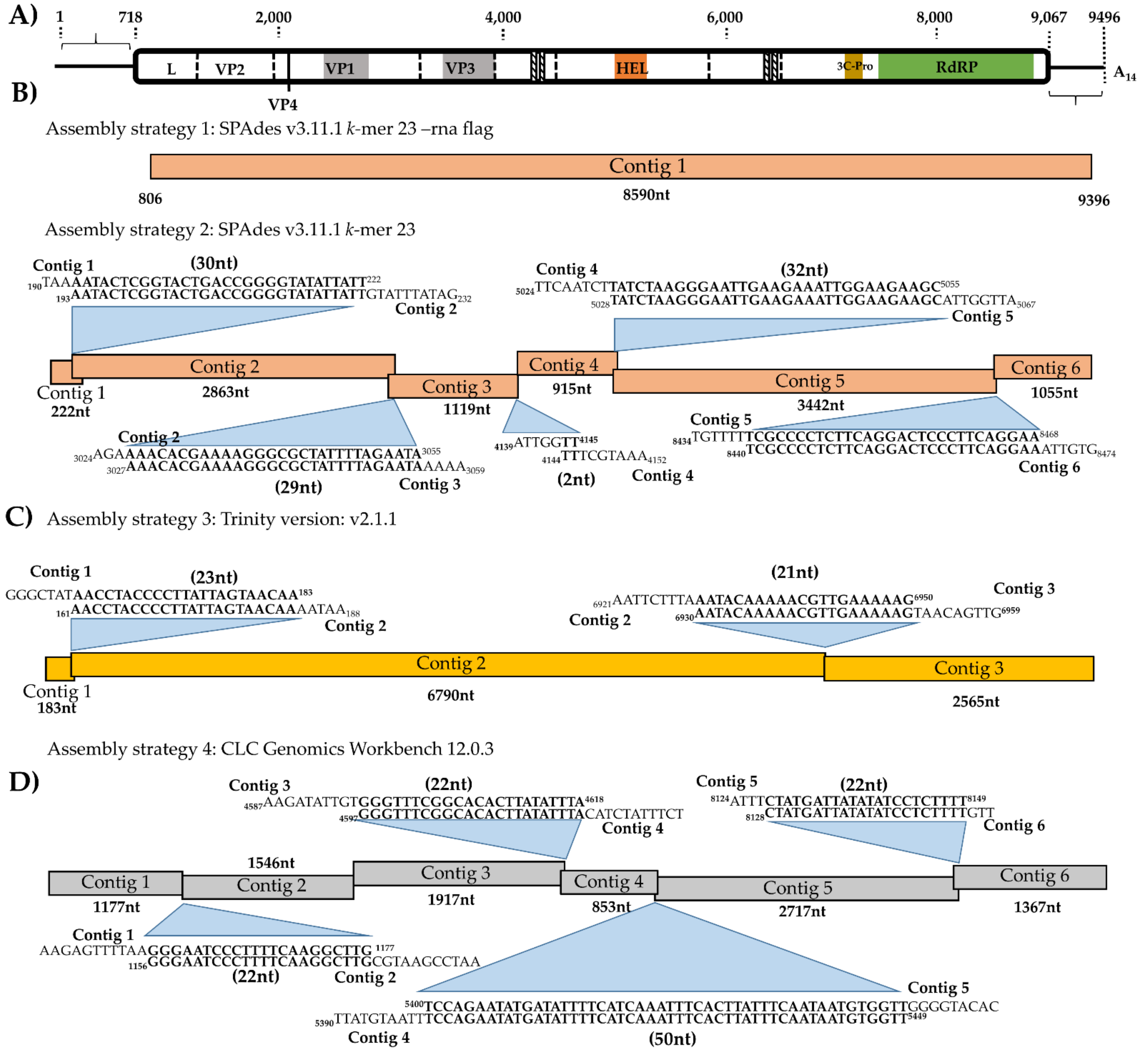

References

1. Bankevich, A.; Nurk, S.; Antipov, D.; Gurevich, A. A.; Dvorkin, M.; Kulikov, A. S.; Lesin, V. M.; Nikolenko, S. I.; Pham, S.; Prjibelski, A. D.; Pyshkin, A. V.; Sirotkin, A. V.; Vyahhi, N.; Tesler, G.; Alekseyev, M. A.; Pevzner, P. A., SPAdes: A new genome assembly algorithm and its applications to single-cell sequencing. *J. Comput. Biol.* **2012,** 19, (5), 455-477.
2. Grabherr, M. G.; Haas, B. J.; Yassour, M.; Levin, J. Z.; Thompson, D. A.; Amit, I.; Adiconis, X.; Fan, L.; Raychowdhury, R.; Zeng, Q. D.; Chen, Z. H.; Mauceli, E.; Hacohen, N.; Gnirke, A.; Rhind, N.; di Palma, F.; Birren, B. W.; Nusbaum, C.; Lindblad-Toh, K.; Friedman, N.; Regev, A., Full-length transcriptome assembly from RNA-Seq data without a reference genome. *Nat. Biotechnol.* **2011,** 29, (7), 644-652.

Figure S2: **AvIFV 5′ UTR Output generated by IRESPred server**

Method: Support vector machine # Server: http://196.1.114.46:1800/IRESPred/Home.html

© Bioinformatics Centre, Savitribai Phule Pune University (formerly University of Pune), Pune, India

Job ID:	1510183876401	s18:	0.95
Sequence Name:	AvIFV 5′	s19:	0.95
Predicted class:	1	s2:	0.9
IRES Prediction:	Potential	s20:	0.9
Sequence Length:	999	s23:	0.9
No. of AUG :	22	s24:	0.95
External loop No.:	1	s26:	0.9
Internal loop No.:	263	s27:	0.85
Hairpin loop No.:	23	s27a:	0.95
Multi loop No.:	10	s28:	0.95
Total Loops:	297	s29:	0.85
Free Energy:	-260.80	s3:	0.85
s11:	0.9	s30:	0.9
s12:	0.85	s3a:	0.9
s13:	1	s4:	0.9
s14:	0.95	s6:	0.9
s15:	0.85	s7:	0.9
s15a:	0.9	s8:	0.9
s16:	0.95	s9:	1
s17:	0.9		

Secondary Structure: Minimum free energy prediction computed using RNAfold 2.4.13 and visualised using the PseudoViewer Web Application (version 3) (http://pseudoviewer.inha.ac.kr/)

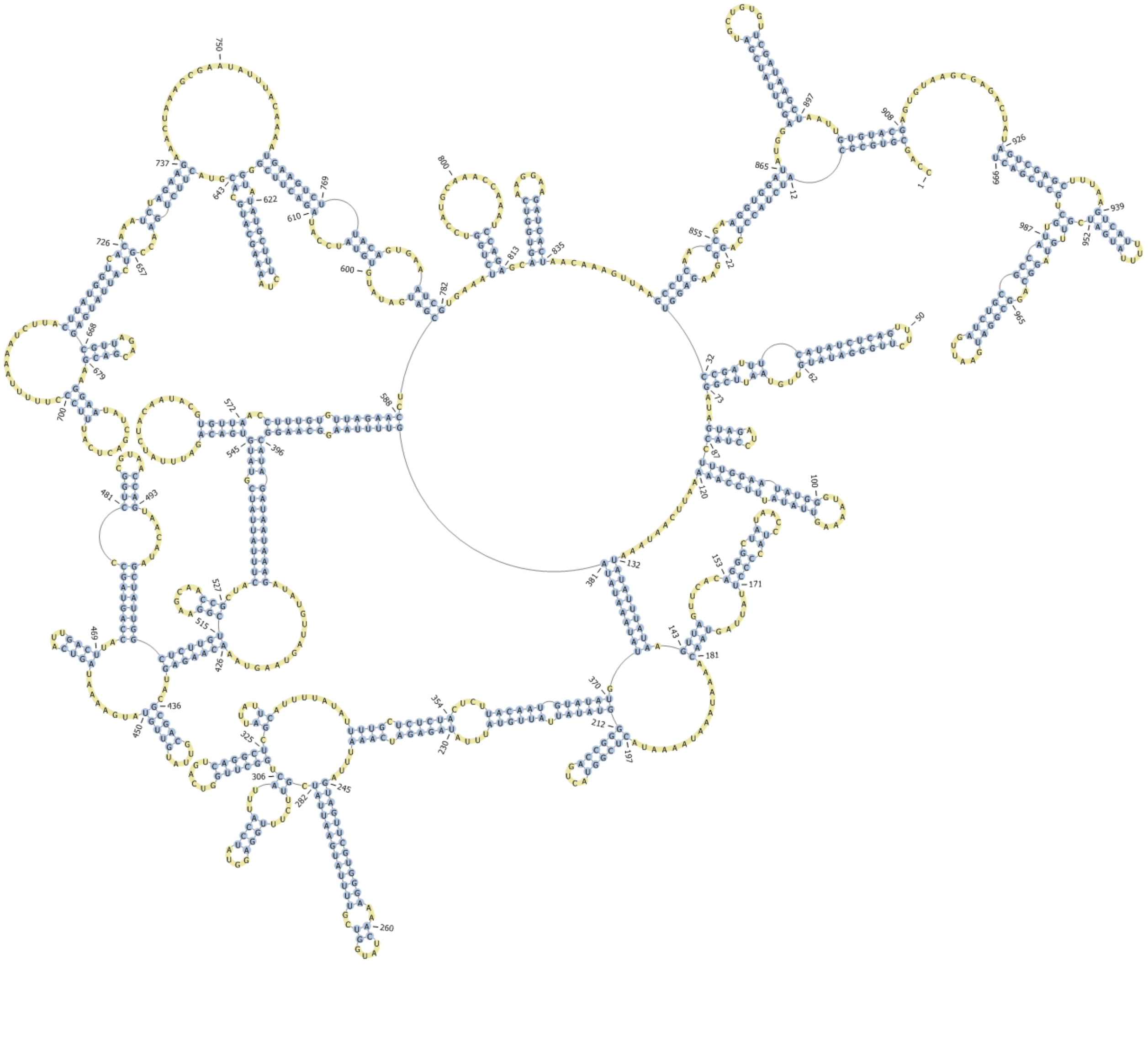

Figure S3: **Amino acid sequence GenBank numbers of picorna(-like) viruses used in domain alignments.** Alignments produced using MUSCLE.

Virus		Genbank accession number
**Family *Iflaviridae***		
**VcPLV**	Venturia canescens picorna-like virus	AAS37668.1*
**HaIV**	Helicoverpa armigera iflavirus	YP_009344960.1
**DcPV**	Dinocampus coccinellae paralysis virus	YP_009111311.1
**NvV**	Nasonia vitripennis virus	ACN94442.1*
**DWV**	Deformed wing virus	APP91308.1
**IFV**	Infectious flacherie virus	NP_620559.1
**SBV**	Sacbrood virus	AID58096.1
**VDV-1**	Varroa destructor virus-1	AGO86045.1
**BIV**	Bat iflavirus	YP_009345906.1
**WiV-13**	Wuhan insect virus 13	YP_009342321.1
**HPLV-30**	Hubei picorna-like virus 30	YP_009337722.1
**WcV-1**	Wuhan coneheads virus 1	YP_009342053.1
**Family *Dicistroviridae***		
**DCV**	Drosophila C virus	NP_044945.1
**CpRV**	Cricket paralysis virus	NP_647481.1
**BQCV**	Black queen cell virus	NP_620564.1
**ABPV**	Acute bee paralysis virus	NP_066241.1
**Family *Picornaviridae***		
**HAV**	Hepatitis A virus	AAA45472.1
**EMCV**	Encephalomyocarditis virus	AAA43037.1
**PV**	Polio virus	CAA24465.1

* Represents a partial sequence of the RNA-dep RNA polymerase

Helicase: Aligned AvIFV polyprotein position 1463-1607. Sequence conservation is indicated by colouring 0% (blue) 50% (black) and 100% (red).

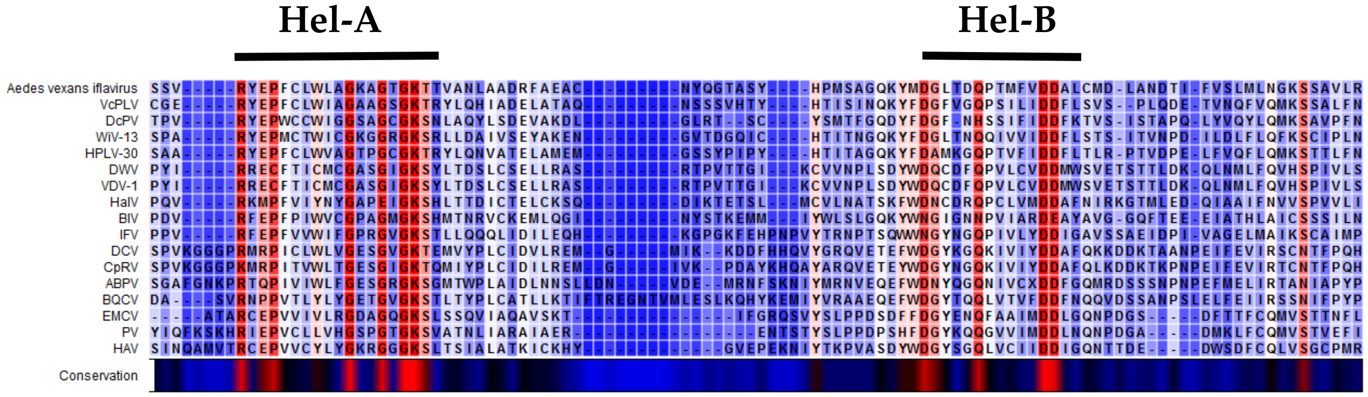

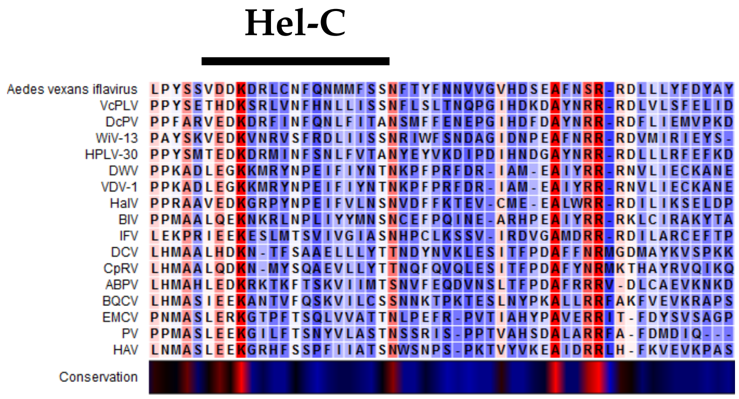

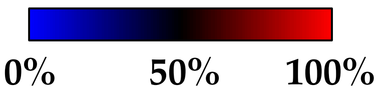

RNA dependent RNA polymerase: Aligned AvIFV polyprotein position 2375-2758. Sequence conservation is indicated by colouring 0% (blue) 50% (black) and 100% (red).

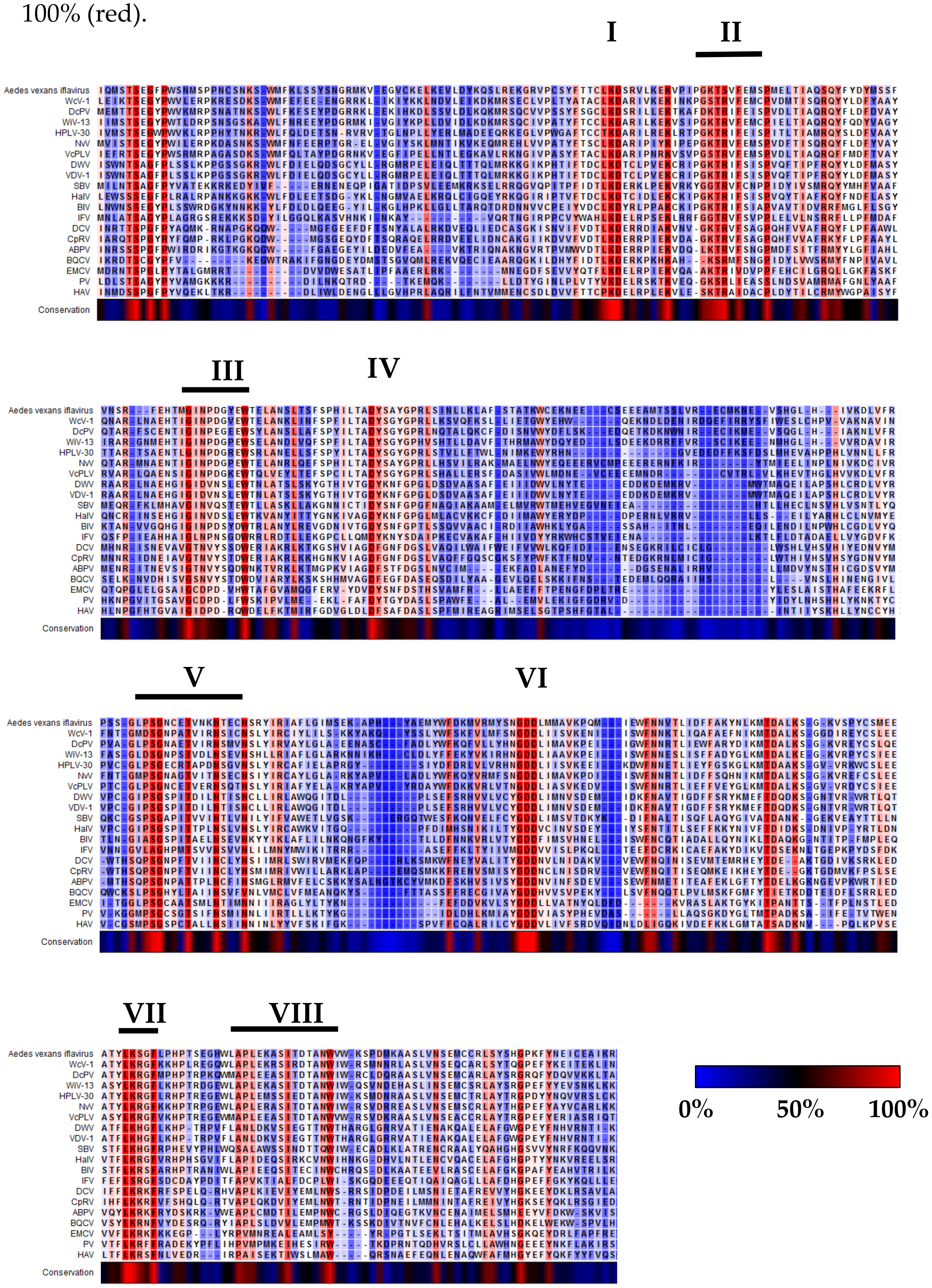

Protease: Aligned AvIFV polyprotein position 2172-2245. Sequence conservation is indicated by colouring 0% (blue) 50% (black) and 100% (red).

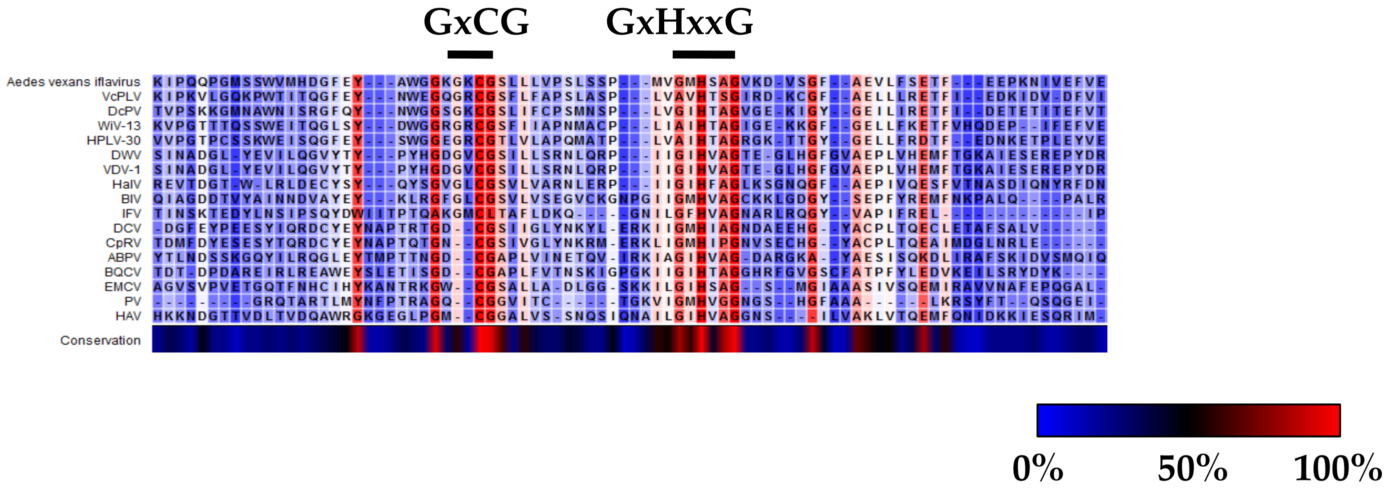

Figure S4: **Agarose gel electrophoretic analysis of RT-PCR and PCR of AvIFV.** A) RT-PCR primers testing for AvIFV gRNA in *Ae. vexans arabiensis* pools from Barkedji, Senegal. B) *Ae. vexans* spp actin host control gene used as an RNA integrity marker. The ladder is GeneRuler 100 bp DNA ladder molecular size marker (Thermofisher™ Scientific, Waltham, MA, US). pEX-AvIFV: corresponds to template DNA originating from pEX-A128-AvIFV plasmid containing synthesised 290bp AvIFV genome fragment. T7-AvIFV corresponds to AvIFV fragment amplified with T7 polymerase ends. Actin DNA control: *Ae vexans* extracted gDNA.

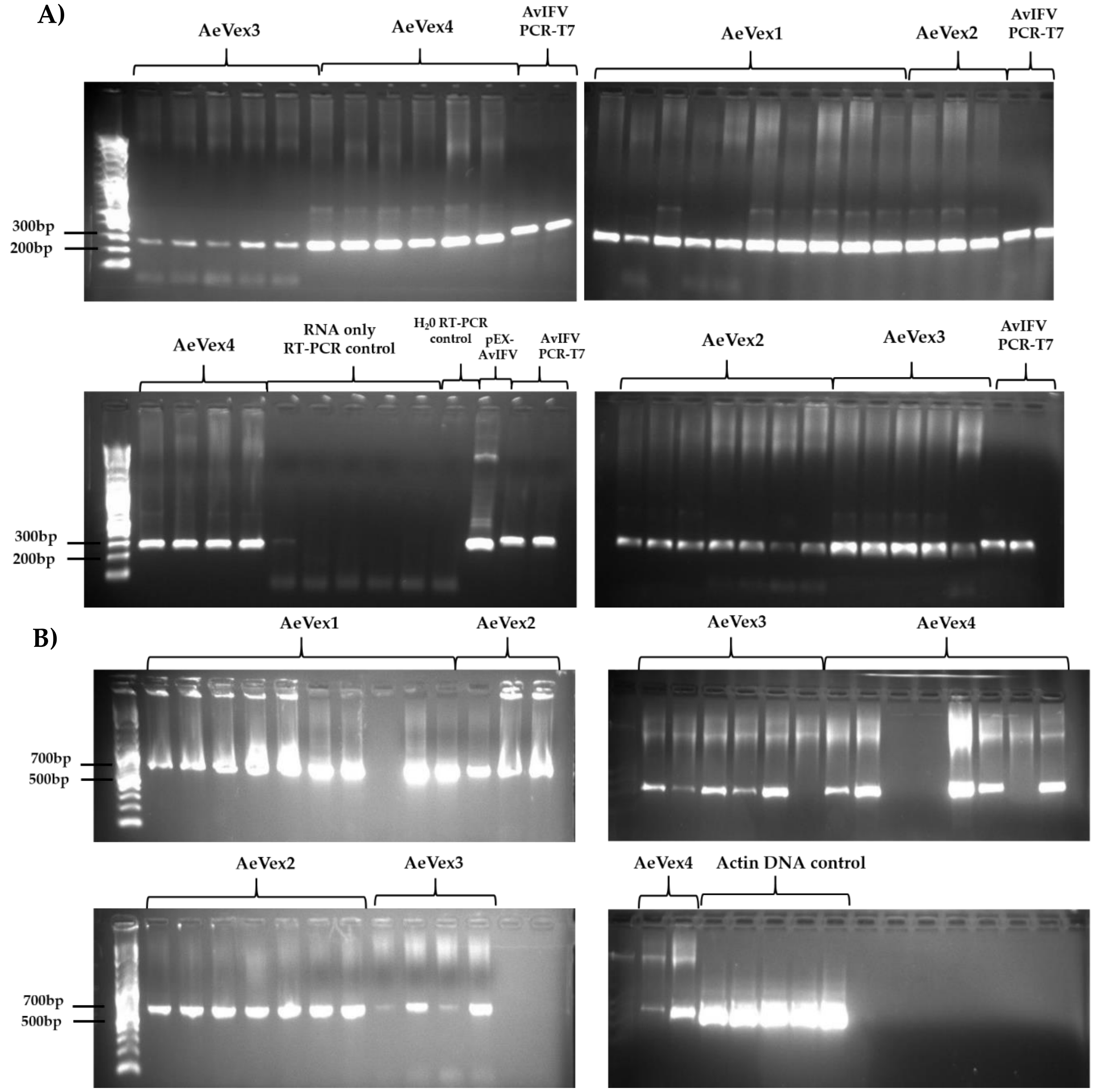

Supplement: Supplementary file 1 [file viruses-12-00440-s001.pdf]
